# Myocardial dysfunction in malnourished children

**DOI:** 10.4103/0974-2069.74036

**Published:** 2010

**Authors:** Nagla Hassan Abu Faddan, Khalid Ibrahim El Sayh, Hamdy Shams, Hosni Badrawy

**Affiliations:** Department of Pediatrics, Egypt; 1 Department of Cardiology, Faculty of Medicine Egypt; 2 Department of Clinical Pathology, South Egypt Cancer Institute, Assiut University, Egypt

**Keywords:** cTnT, echocardiography, protein energy malnutrition

## Abstract

**Background:**

Malnourished children suffer several alterations in body composition that could produce cardiac abnormalities.

**Aim:**

The aim of the present study was to detect the frequency of myocardial damage in malnourished children as shown by echocardiography and cardiac troponin T (cTnT) level.

**Methods:**

Forty-five malnourished infants and young children (mean±SD of age was 11.24 ±7.88 months) were matched with 25 apparently healthy controls (mean±SD of age was 10.78±6.29 months). Blood sample was taken for complete blood picture, liver and kidney function tests, serum sodium, potassium, calcium levels and cTnT. All the malnourished children were subjected to echocardiographic evaluation.

**Results:**

Malnourished children showed a significantly lower left ventricular (LV) mass than the control group. The LV systolic functions were significantly impaired in patients with severe malnutrition. The cTnT level was higher than the upper reference limits in 11 (24.44%) of the studied malnourished children and all of them had a severe degree of malnutrition. The cTnT level was significantly higher in patients with anemia, sepsis and electrolyte abnormalities and it correlated negatively with LV ejection fraction (EF). Six of the studied children with high cTnT levels (54.5%) died within 21 days of treatment while only one case (2.9%) with normal level of cTnT died within the same period.

**Conclusions:**

LV mass is reduced in malnourished children. Children with severe malnutrition have a significant decrease in LV systolic functions. Elevated cTnT levels in malnourished children has both diagnostic and prognostic significance for cardiomyocyte damage.

## INTRODUCTION

Protein-energy malnutrition (PEM) can be defined as the state of decreased body pools of protein with or without fat depletion that is caused, at least in part, by inadequate nutrient intake relative to nutrient demand that is needed to ensure growth and maintenance.[1,2] PEM affects approximately one-third of children worldwide and is frequently seen in less-developed countries due to inadequate food intake, socioeconomic factors or, at times, due to natural disasters.[3,4] Thus, PEM is the concern of many researches.[5] Malnourished children suffer several alterations in body composition, with loss of heart and skeletal muscle mass, complicated by electrolyte abnormalities and mineral or vitamin deficiencies that could produce cardiac abnormalities, including hypotension, cardiac arrhythmias, cardiomyopathy, cardiac failure and even sudden death.[3,6] Cardiac troponins are regulatory proteins of the thin actin filaments of the cardiac muscle.[7] Myocardial cell injury results in the release of cardiac troponin, which differs from troponin isoforms of the skeletal muscle, and thus is a highly sensitive and specific biomarker of myocardial damage.[8] It is the best known molecular marker of myocardial injury.[9] Mortality among troponin-positive patients was reported to be higher compared with that among troponin-negative patients, irrespective of the cause of troponin positivity.[10] A number of studies have suggested a possible association between cardiac troponin and myocardial injury in patients with non-cardiac diseases.[11–14] Several authors reported a relationship between elevated cardiac troponin levels and LV dysfunction, assessed by echocardiography.[11,15,16]

The aim of this study was to detect the frequency of myocardial damage among malnourished children as shown by echocardiography and cardiac troponin T (cTnT) levels.

## METHODS

The current study included 45 malnourished infants and young children diagnosed according to the Waterlow classification (1972).[17] They were recruited from the Inpatient Department of Assiut University Children Hospital, Egypt. Their ages ranged from 2 to 30 months. There were 25 boys and 20 girls. Three children had first-degree marasmus (M I), 16 had second-degree marasmus (M II), 19 had third-degree marasmus (M III), four had kwashiorkor (KWO) and three had marasmic-kwashiorkor (M-KWO).

Exclusion criteria were the following:

- Pre-term infants or intrauterine growth retardation at birth.
- Any documented cardiothoracic event (congenital heart disease, pericarditis, cardiomyopathy, acute severe lower respiratory tract infection, etc.).
- Severe anemia (blood hemoglobin level ≤6 g/dl).

Twenty-five apparently healthy infants and young children were studied as a control group. Their age ranged from 4 to 24 months. They were 11 boys and 14 girls. After obtaining approval of ethical committee of the Faculty of Medicine, Assuit University, an informed written consent was taken from the parents or legal guardians. Before nutritional rehabilitation, all studied malnourished infants were subjected to full history taking, including dietetic history, thorough clinical examination, with special emphasis on the anthropometric measurements and signs of malnutrition. The reference value of height and weight were compared to the growth charts of Cairo University.[18] Patients and controls were subjected to the following investigations:

- Complete blood picture.
- Liver and kidney function tests.
- Serum sodium, potassium and calcium levels.
- Assessment of cTnT by electrochemiluminescence immunoassay (ECLIM) I intended for use on the Roche Elecsys 1010 immunoassay analyzer. The lower detection limit was 0.010 ng/ml.[19] Values below the detection limit are reported as <0.010 ng/ml.

### Echocardiographic evaluation

All patients underwent transthoracic echocardiographic examination using the Hewlett Packard (HP) sonos 4500 using phased array transducers with a frequency of 8 MHZ. Imaging was performed with the patient in a recumbent or lateral decubitus position. M-mode echo is a standard method for assessment of LV function in the absence of segmental wall motion abnormalities,[20,21,22] which was the case in the present study, and therefore M-mode, 2-dimensional echocardiography, pulsed and continuous wave Doppler and color flow mapping were performed in every patient using the standard views as parasternal long axis, short axis, apical four, five chamber and subcostal views to assess the following parameters:

1. LV function
  - LV dimensions: measured from the derived M-mode echocardiography in the parasternal long axis view. All the tracings were recorded using the leading-edge technique.[23]
  - Percentage of fractional shortening (FS): LV FS was calculated using the following formula:
    FS=EDD-ESD/EDD×0;100[24]
    where, EDD is the end diastolic diameter of the left ventricle and ESD is the end systolic diameter of the left ventricle
  - Ejection fraction (EF): measured from the “cubed equation”:
    EF=(EDD)-(ESD)/(EDD)×100
  - LV diastolic frunction (E/A): using E/A ratio of the mitral flow by pulsed wave Doppler across the mitral valve.
2. Left ventricular mass (LVM)and left ventricular mass index(LVMI) It is performed using the LVMI calculator.

Nutritional rehabilitation was done in all patients according to the WHO (1999) recommendation.[25]

### Statistical analysis

Analysis was carried out using SPSS (version 16). The numerical data were represented as mean±SD. For comparison of the two groups, Student’s *t*-test was used for parametric data and the Mann–Whitney *U*-test was used for non-parametric data. Multiple groups were compared using the ANOVA test. Linear correlations were performed by Spearman’s or Pearson’s test. For all tests, the difference was considered significant if the probability (*P*) was <0.05

## RESULTS

Table 1 shows some clinical and laboratory data of the studied patients compared with the control group. No statistically significant difference between patients and controls regarding the heart rate, the mean diastolic and systolic blood pressure, liver and kidney functions could be detected. Table 2 shows the echocardiographic findings of patients with PEM compared with the controls. The cTnT level and the echocardiographic findings of different groups of malnutrition and controls are shown in Table 3.

**Table 1:** Clinical and laboratory data of the studied patients with PEM compared with controls

Variant	Age (months)	Weight (kg)	Height (cm)	BMI (wt/ht^2)^	Na (m eq/l)	K (m eq/l)	Ca (mg/dl)	Total protein (g/dl)	Albumin (g/dl)	Hg (g/dl)	WBCs (×10^3^/μl)	cTnT (ng/ml)
Patients (*n*=45)	11.24±7.88	5.4±1.6	63.4±8.93	13.33±2.19	137.4±5.23	3.82±0.54	8.64±1.25	61.43±9.68	34.38±8.4	9.39±1.6	10.52±4.23	0.01±0.01
Controls (*n*=25)	10.78±6.29	8.97±2.26	74.8±9.54	15.81±2.28	139.64±3.1	3.88±0.454	9.15±1.0	70.61±7.34	40.14±11.91	12.53±1.1	8.83±3.6	0.009±0.001
*P*-value	NS	<0.005*	<0.005*	<0.005*	NS	NS	<0.005*	<0.005*	0.025*	<0.005*	<0.005*	0.007*

PEM, Protein–energy malnutrition; BMI, body mass index; Na, serum sodium; K, serum potassium; Ca, serum calcium; Hg, hemoglobin level; WBCs, total leucocytic count; cTnT, cardiac troponin T. Quantitative variables are expressed as mean±standard deviation. Student’s *t*-test was used.

* statistically significant result. NS, non-significant result

**Table 2:** Echocardiographic findings of the patients with PEM compared with the controls

Echo	LVSD (cm)	EDD (cm)	ESD (cm)	PWD (cm)	LVM (g)	LVMI (g/m^2^)	FS (%)	EF (%)	E/A
Patients (45)	0.40±0.05	2.56±0.63	1.55±0.42	0.4±0.08	18.96±7.59	67.78±23.04	0.46±0.13	40.31±9.12	72.6±10.76
Controls (25)	0.46±0.05	2.84±0.52	1.72±0.36	0.45±0.07	29.89±12.09	64.46±12.59	0.57±0.12	55.46±22.04	75.09±8.54
*P*-value	<0.005*	NS	NS	0.013*	<0.005*	NS	0.001*	<0.005*	NS

PEM, Protein –energy malnutrition; LVSD, left ventricular septal diameter; EDD, end diastolic diameter of left ventricle; ESD, end systolic diameter of left ventricle; PWD, posterior wall diameter; FS, fractional shortening; EF, ejection fraction; LVM, left ventricular mass; LVMI, left ventricular mass index; E/A ratio, E wave/A wave ratio. Quantitative variables are expressed as mean±standard deviation. Student’s *t*-test was used.

* statistically significant result. NS, non-significant result

**Table 3:** Cardiac troponin T and echocardiographic data of different groups of malnutrition and controls

	cTnT (ng/ml)	LVSD (cm)	EDD (cm)	ESD (cm)	PWD (cm)	LVM (g)	LVMI (g/m^2^)	FS (%)	EF (%)	E/A
(1) = MI+ MII (19/45)	0.093±0.004	0.41±0.02	2.8±0.71	1.68±0.45	0.39±0.51	22.73±8.86	72.39±1.66	0.52±0.13	45.63±8.05	77.15±8.85
(2) = MIII (19/45)	0.138±0.01	0.39±0.06	2.41±0.53	1.46±0.37	0.39±0.07	17.3±4.96 A	69.93±2.59	0.46±0.13	38.73±6.83 A	71±7.44 A
(3) = KWA + M-KWA (7/45)	0.028±0.018A, B	0.35±0.02 A	2.27±0.55 A	1.4±0.43	0.35±0.018	13.19±4.09 A	45.24±1.93 A	0.45±0.97	30.14±7.53 A, B	64.57±1.72 A
(4) = Controls (25)	0.093±0.006 B, C	0.46±0.05 A, B, C	2.84±0.52 B, C	1.72±0.36 B, C	0.45±0.07 A, B, C	29.89±12.09 A, B, C	64.46±1.25 A, B, C	0.57±0.12 B, C	55.46±2.204 B, C	75.09±8.54 C

cTnT, cardiac troponin T; LVSD, left ventricular septal diameter; EDD, end diastolic diameter of left ventricle; ESD, end systolic diameter of left ventricle; PWD, posterior wall diameter; FS, fractional shortening; EF, ejection fraction; LVM, left ventricular mass; LVMI, left ventricular mass index; E/A ratio, E wave/A wave ratio. Quantitative variables are expressed as mean×standard deviation. ANOVA test was used. *statistically significant result. NS, non-significant result. A = significant result with group 1. B = significant result with group 2. C = significant result with group 3

A weak insignificant positive correlation was found between either LVM or LVMI and the body mass index (BMI) (r=0.128, P=0.403 and r=0.126 and P=0.411, respectively).

**Figure 1:**
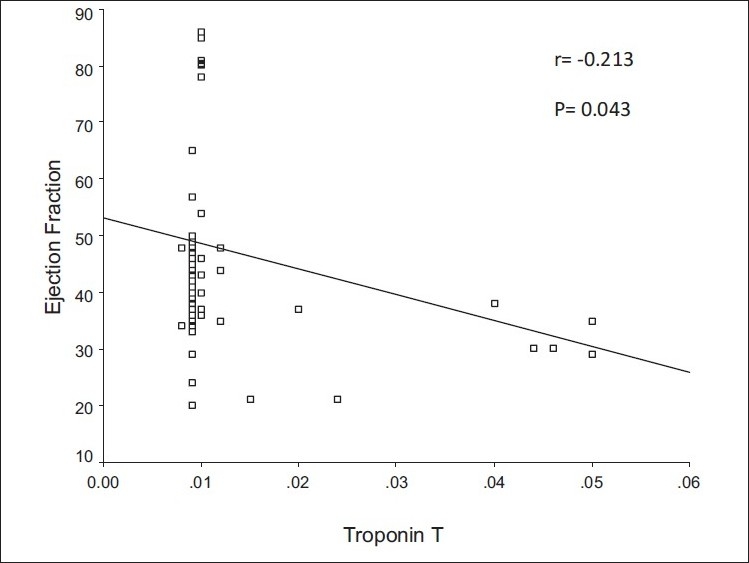
Correlation between cTnT and LVEF, cTnT, cardiac troponin T; LVEF, Left ventricular ejection fraction

In the present study, the cTnT levels were higher than the upper reference limits in 11 of the studied malnourished children (24.44%) and all of them had a severe degree of malnutrition; six (54.5%) had M III, two (18%) had KWO and three (27.7%) had M-KWO. Table 4 shows some laboratory and echocardiographic parameters of malnourished children with normal cTnT levels compared with those with high cTnT levels. The cTnT level was significantly higher in patients with anemia, electrolyte deficiency and sepsis. Figure 1 shows a significant negative correlation between cTnT level and LVEF, r=-0.213, P=0.043. Six of the studied children (54.5%) with high cTnT levels died within 21 days of treatment while only one case (2.9%) with baseline level of cTnT died within the same period. Table 5 shows some laboratory and echocardiographic parameters in the studied patients with PEM according to the outcome.

**Table 4:** Laboratory and echocardiographic parameters of the studied patients with normal cTnT levels compared with those with high cTnT levels

Variant	Na (m eq/l)	K (m eq/l)	Ca (mg/dl)	Total protein (g/dl)	Albumin (g/dl)	Hg (g/dl)	WBCs (×lO^3^/μl)	FS (%)	EF (%)	E/A	Mortality %
Patients with normal cTnT (*n*=34)	138.3±4.95	3.9±0.48	8.8±1.14	63.32±7.7	36.1±7.7	9.85±1.12	9.97±4.19	48.24±12.17	42.52±8.27	73.88±8.96(2.94)	1/34
Patients with high cTnT (*n* =11)	134.6±5.32	3.5±0.63	8.1±1.45	55.59±8.7	29.1±8.6	7.96±2.01	15.21±4.04	50.91±14.46	33.45±8.45	68.63±1.49	6/11(54.54)
*P*value	0.037*	0.062*	NS	0.004*	0.006*	0.001*	0.014*	NS	0.004*	NS	<0.005*

Na, serum sodium; K, serum potassium; Ca, serum calcium; Hg, hemoglobin level; WBCs, total leucocytic count; FS, fractional shortening; EF, ejection fraction; E/A ratio, E wave/A wave ratio. Quantitative variables are expressed as mean±standard deviation. Mann–Whitney *U*-test was used. Fisher exact test.

* statistically significant result. NS, non-significant result

**Table 5:** Laboratory and echocardiographic parameters in studied patients with PEM according to the outcome

Variant	Na (m eq/l)	K (m eq/l)	Ca (mg/dl)	Total protein (g/dl)	Albumin (g/dl)	Hg (g/dl)	WBCs (x10 V, ul)	cTnT (ng/ml)	FS (%)	EF (%)	E/A
Survivors (*n*=38)	137.97±4.79	3.94±0.47	8.74±1.2	62.7±9.6	35.55±7.7	9.5±1.64	10.3±4.2	0.01±0.00	49.2±13.2	42.31±8.1	74.3±9.1
Non-survivors (*n*=7)	134.4±6.8	3.18±0.44	8.08±1.31	54.57±9.7	28.01±9.7	8.7±1.17	11.6±4.4	0.03±0.01	47.1±0.1	29.4±6.8	63.4±1.5
*P* value	NS	0.001*	NS	0.026*	0.047*	NS	NS	<0.005*	NS	0.001*	NS

PEM, Protein –energy malnutrition; Na, serum sodium; K, serum potassium; Ca, serum calcium; Hg, hemoglobin level; WBCs, total leucocytic count; cTnT, cardiac troponin T; FS, fractional shortening; EF, ejection fraction; E/A ratio, E wave/A wave ratio. Quantitative variables are expressed as mean×standard deviation. Mann–Whitney *U*-test was used

* statistically significant result

## DISCUSSION

PEM is a serious disease responsible for high morbidity and mortality rates among children in developing countries.[26] Children suffering from severe malnutrition frequently exhibit cardiovascular abnormalities.[3] Echocardiographic evaluation of children with PEM in the present study revealed a significantly lower LV septal thickness, posterior wall thickness and LVM than the control group [Table 2]. This decrement was more prominent in patients with M III, KWO and M-KWO [Table 3]. These findings are in agreement with those of previous authors who reported that in patients with PEM, the heart is unable to escape from atrophy affecting other organs.[3,5,6] Cunha *et al*.[27] suggested that the cause of diminished cardiac mass in patients with PEM was slow myocardial anabolic rate rather than increased catabolism. In the present study, we did not find a significant correlation between either LVM or LVMI and BMI. Our data were different from those reported by Olivers *et al*.,[3] who found that the decrement of LVM and LVMI was proportional to the decrement in the total body mass. This difference may be due to a smaller number of patients in the present study. Most investigators agree that the heart atrophies during starvation, but controversy persists as to whether atrophic heart with PEM functions normally or whether it demonstrates LV dysfunction. Also, it was not clear whether there was a difference in cardiac performance as a function of type and severity of PEM.[6] The present study showed that the parameters of the LV systolic function (FS and EF) were significantly affected in patients than in the controls [Table 2], and this affection was more prominent in patients with M III, KWO and M-KWO [Table 3]. This is in agreement with reports from previous authors,[28–30] who reported that LV systolic function was reduced in children with severe cases of PEM with more than 40% loss of the expected weight. Shoukry *et al*.,[31] reported that infants with KWO had a reduction in the FS compared with the controls. Others did not find any evidence of LV systolic dysfunction in their patients.[3,5,6,32] This difference possibly could be due to the effect of other factors such as electrolyte imbalance or trace element deficiency that affect LV systolic function in the different studies. Regarding the LV diastolic function (the E/A ratio), the present study showed no significant difference between patients and the control group [Table 2]. These findings are in keeping with those reported by other studies.[3,5,6]

Myocardial damage in children may be clinically occult in a variety of stressful settings.[33] Measurement of cTnT in blood is considered one of the gold standards for detecting heart damage. Cardiac cTnT is an abundant cardiac protein that is not detected at a significant level in healthy individuals and is released predominantly during cardiac damage.[34] The present study revealed that cTnT levels were higher than the upper reference limits in 11 of the malnourished children (24.44%), all having a severe degree of malnutrition; six of them (54.5%) had M III, two (18%) had KWO and three (27.7%) had M-KWO. The cTnT level was significantly higher in patients with anemia, sepsis and electrolyte deficiency [Table 4]. This is in line with that reported by Elsayed *et al*.,[5] who stated that in patients with PEM, there is no place for cardiac proteins to be released massively in a detectable way in circulation except in acute severe cases or in the presence of complications. A significant negative correlation was found between cTnT level and LVEF [Figure 1]. These findings could be explained by the presence of complications such as sepsis, electrolyte imbalance and anemia, which are usually found in cases of severe PEM, which, either alone or in combination, may cause myocardial injury and, consequently, affect LVEF, causing a significant elevation in cTnT in the blood. Children may have more deleterious consequences of low-level cardiomyocyte loss than adults due to both the length of subsequent survival and an insufficient potential for myocardial growth to compensate for both early damage and somatic development.[35]

The increased cTnT level is not only used as an indicator of myocardial damage but also for prognostic information.[33,35] In the present study, six malnourished children (54.5%) with high cTnT levels died within 21 days of treatment while only one case (2.9%) with baseline level of cTnT died within the same period [Table 5]. These results are in keeping with the results of Kontos *et al*.,[35] who reported that the mortality at 30 days was significantly higher among patients with elevated troponin levels at presentation than among patients with no biomarkers detected. These cardiac changes in patients with PEM denote the severity of the disease and its fatal outcome, requiring urgent effective measures to overcome this serious disease. Nutritional rehabilitation can reverse these abnormalities to a great extent.[5]

## CONCLUSION

LVM is reduced in malnourished children. Children with M III, KWO and M-KWO have a significant decrease in LV systolic functions. Elevated cardiac troponin levels in patients with PEM has both diagnostic and prognostic significance for cardiomyocyte damage.
